# Amyloidosis of lacrimal gland

**DOI:** 10.4103/0301-4738.64137

**Published:** 2010

**Authors:** Vinod Kumar, Neha Goel, Luke Nicholson, Jai Shankar

**Affiliations:** Wrexham Maelor Hospital, Wrexham Wales, UK; 1 Guru Nanak Eye Centre, Maulana Azad Medical College, New Delhi, India

Dear Editor,

We read with interest the article "Amyloidosis of lacrimal gland" by Prabhakaran *et al*.[1] We would like to congratulate the authors for this well-documented case and would like to make these observations.

The authors report that approximately 24 cases of primary localized amyloidosis of orbit have been reported. However, we believe that this number is likely to be higher. In a review article by Taban *et al*.,[2] the authors reviewed 31 cases of primary orbital amyloidosis including one of their own. There have been a few further reports after that including a large series by Leibovitch *et al*.[3]

The authors mention that one bilateral case of isolated lacrimal gland amyloidosis has been reported. We wish to draw attention to one of the cases reported by Cheng *et al*.,[4] who had bilateral isolated lacrimal gland involvement with amyloidosis. Also, Knowles *et al*.,[5] have described a case with serial bilateral lacrimal gland amyloidosis without systemic disease (primary localized orbital amyloidosis). This raises the total number of cases previously reported to have bilateral isolated lacrimal gland amyloidosis to at least three.

Hertel's exophthalmometer is designed to measure axial proptosis and is unable to measure ocular displacement. We would value being educated as to how the authors were able to measure the displacement with the help of a Hertel's exophthalmometer. Perhaps the authors are merely implying that apart from having 2 mm of axial proptosis, the patient also had 2 mm of displacement.

Lastly, was any reoccurrence noted? Recurrence has been said to occur in approximately one-third of cases as total excision is usually not possible in these patients.
